# Supplementary material for: Methionine Cycle Rewiring by Targeting miR-873-5p Modulates Ammonia Metabolism to Protect the Liver from Acetaminophen
Source: Antioxidants (Basel). 2022 Apr 30;11(5):897. doi: 10.3390/antiox11050897 (PMC9137496; doi:10.3390/antiox11050897)
Supplement: Supplementary file 1 [file antioxidants-11-00897-s001.zip › antioxidants-1673473-supplementary.pdf]

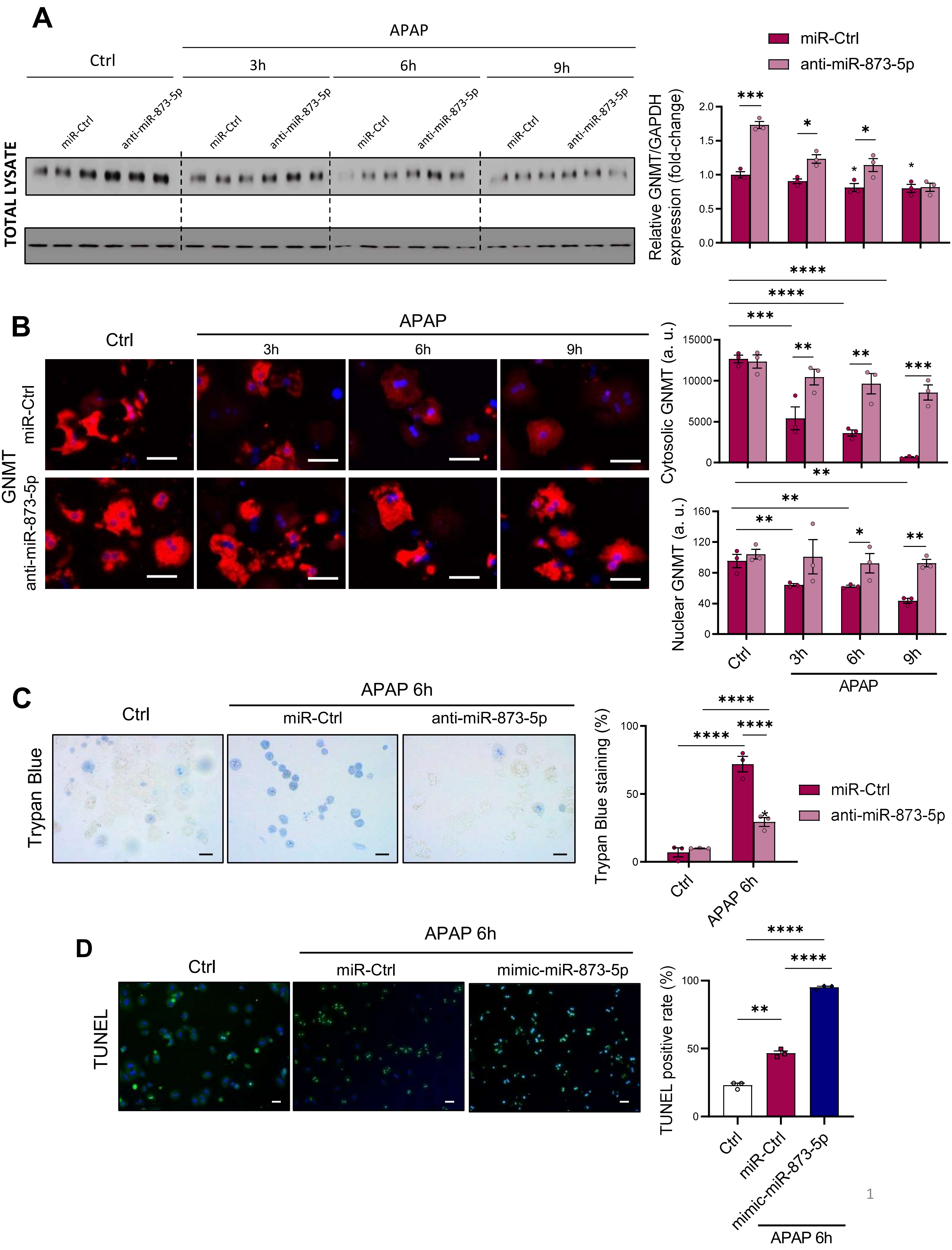
A
APAP
Ctrl
3h
6h
9h
miR-Ctrl
anti-miR-873-5p
TOTAL LYSATE
Relative GNMT/GAPDH expression (fold-change)
B
GNMT
Cytosolic GNMT (a. u.)
Nuclear GNMT (a. u.)
C
APAP 6h
Trypan Blue
Trypan Blue staining (%)
D
mimic-miR-873-5p
TUNEL
TUNEL positive rate (%)

Figure S1. miR-873-5p-dependent glycine N-methyltransferase expression and hepatotoxicity. GNMT determination in primary hepatocytes treated with different times (3, 6 and 9 hours) of 10 mM acetaminophen (APAP) and treated with an anti-miR-873-5p or an unrelated control (miR-Ctrl) by **A.** Western blot (GAPDH was used as a loading control to perform relative quantification) and **B.** Immunofluorescence and respective determination for cytoplasmic and nuclear staining; **C.** Representative micrographs and respective determination of Tryplan Blue staining in primary wild-type hepatocytes treated with 10 mM APAP for 6 h and transfected with anti-miR-873-5p or miR-Ctrl. **D.** Representative micrographs and respective determination of TUNEL in primary wild-type hepatocytes treated with 10 mM APAP for 6 h and transfected with a mimic-miR-873-5p or miR-Ctrl Scale bar corresponds to 50 µm. *p<0.05, **p<0.01, ***p<0.001, ****p<0.0001 are shown (Related to Figure 3).

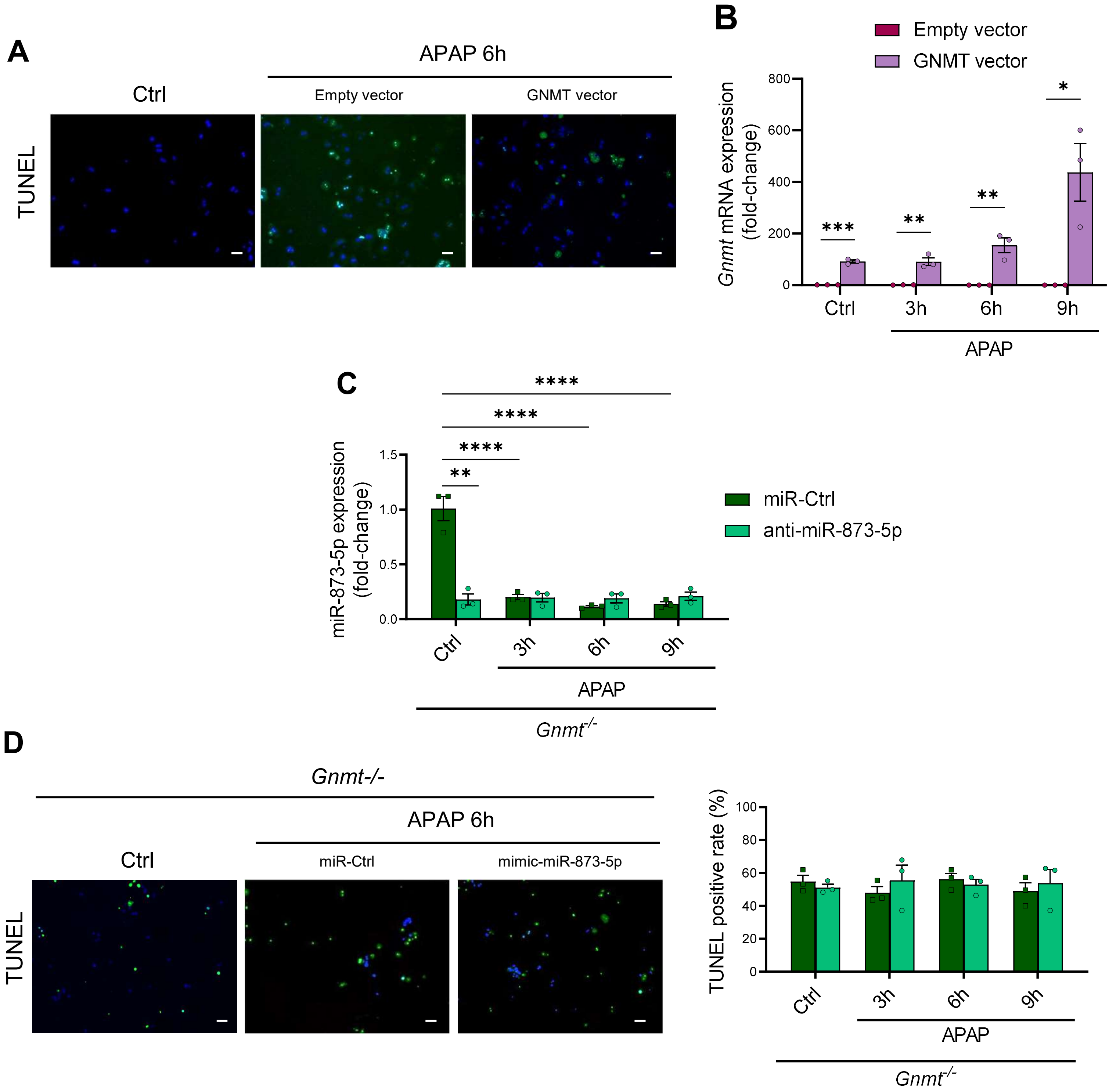

Figure S2. mRNA expression of *Glycine N-methyltransferase* (*Gnmt*) by transient transfection in wild-type hepatocytes and miR-873-5p expression and hepatotoxicity in primary hepatocytes lacking *Gnmt.* **A.** Representative micrographs at 6 h APAP and **B.** Relative *Gnmt* expression in primary wild-type hepatocytes treated with APAP for 3, 6 and 9 h and transfected with an empty or GNMT expression vector; **C.** Relative miR-873-5p expression and **D.** TUNEL determination in primary hepatocytes from *Gnmt*$^{-/-}$ mice cultured for 3, 6 and 9 h under 10 mM APAP and treated with an anti-miR-873-5p or miR-Ctrl; **p*<0.05, ***p*<0.01, ****p*<0.001, *****p*<0.0001 are shown. (Related to Figure 3)

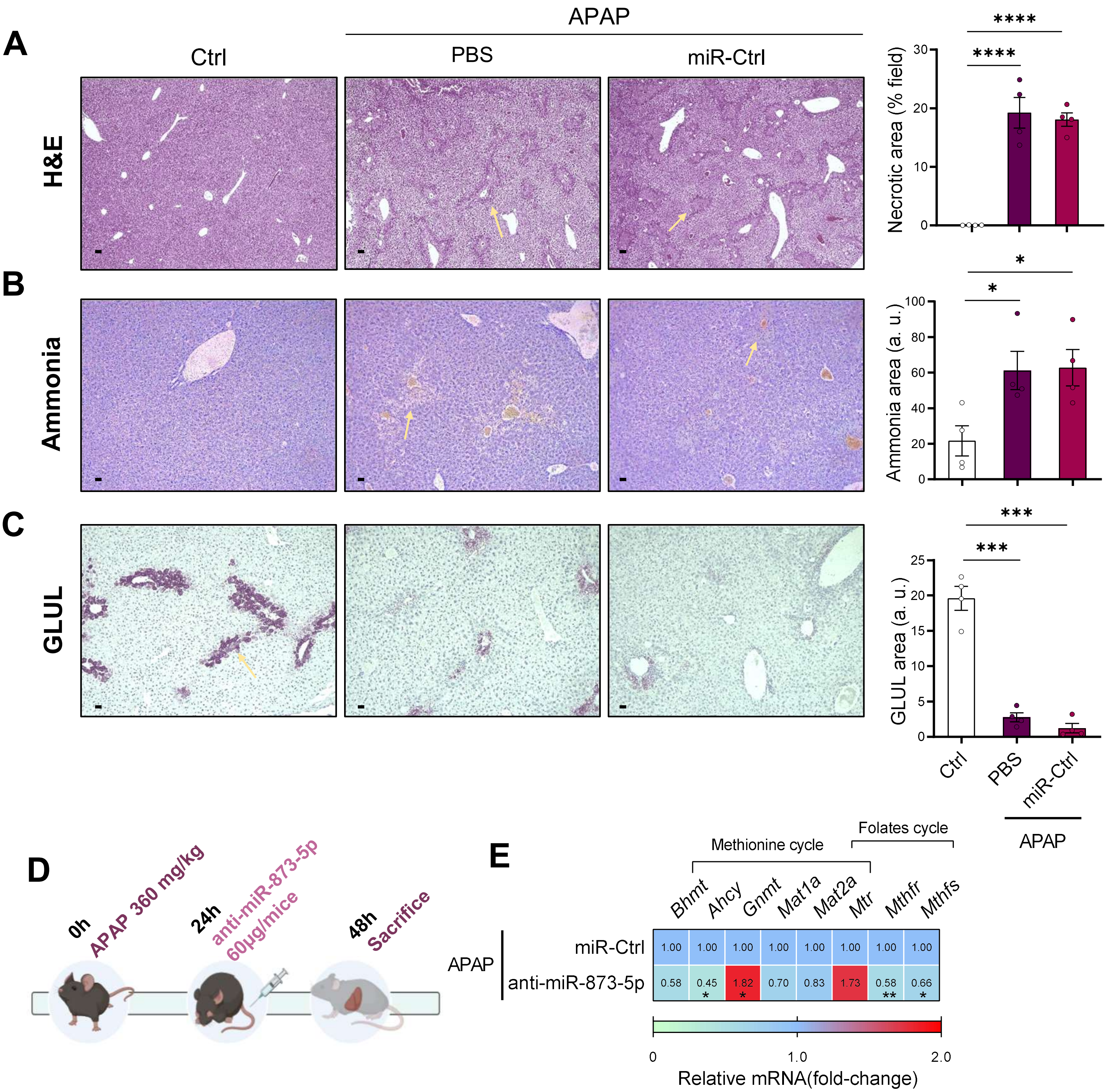

		Bhmt	Ahcy	Gnmt	Mat1a	Mat2a	Mtr	Mthfr	Mthfs
APAP	miR-Ctrl	1.00	1.00	1.00	1.00	1.00	1.00	1.00	1.00
	anti-miR-873-5p	0.58	0.45 *	1.82 *	0.70	0.83	1.73	0.58 **	0.66 *

Figure S3. Histopathological characterization of miR-Ctrl versus vehicle, schematic representation of preclinical study and mRNA expression array from methionine and folates cycle. Representative micrographs and respective determination of **A.** hepatic necrotic áreas, **B.** ammonia staining and **C.** glytamine synthase (GLUL) in liver from mice treated with 360 mg/kg APAP for 48 h and either PBS or a miRNA against an unrelated control (miR-Ctrl). Scale bar corresponds to 50 µm and stainings are highlighted in yellow arrow. **D.** Schematic represetation of pre-clinical study in mice by treating them APAP and injecting an anti-miR-873-5p or miR-Ctrl through tail vein injection; **E.** Relative mRNA expression of folates cycle and methionine cycle enzymes in liver from mice treated with APAP and miR-Ctrl or anti-miR-Ctrl. *p<0.05, **p<0.01, ***p<0.001, ****p<0.0001 . (Related to Figure 3).

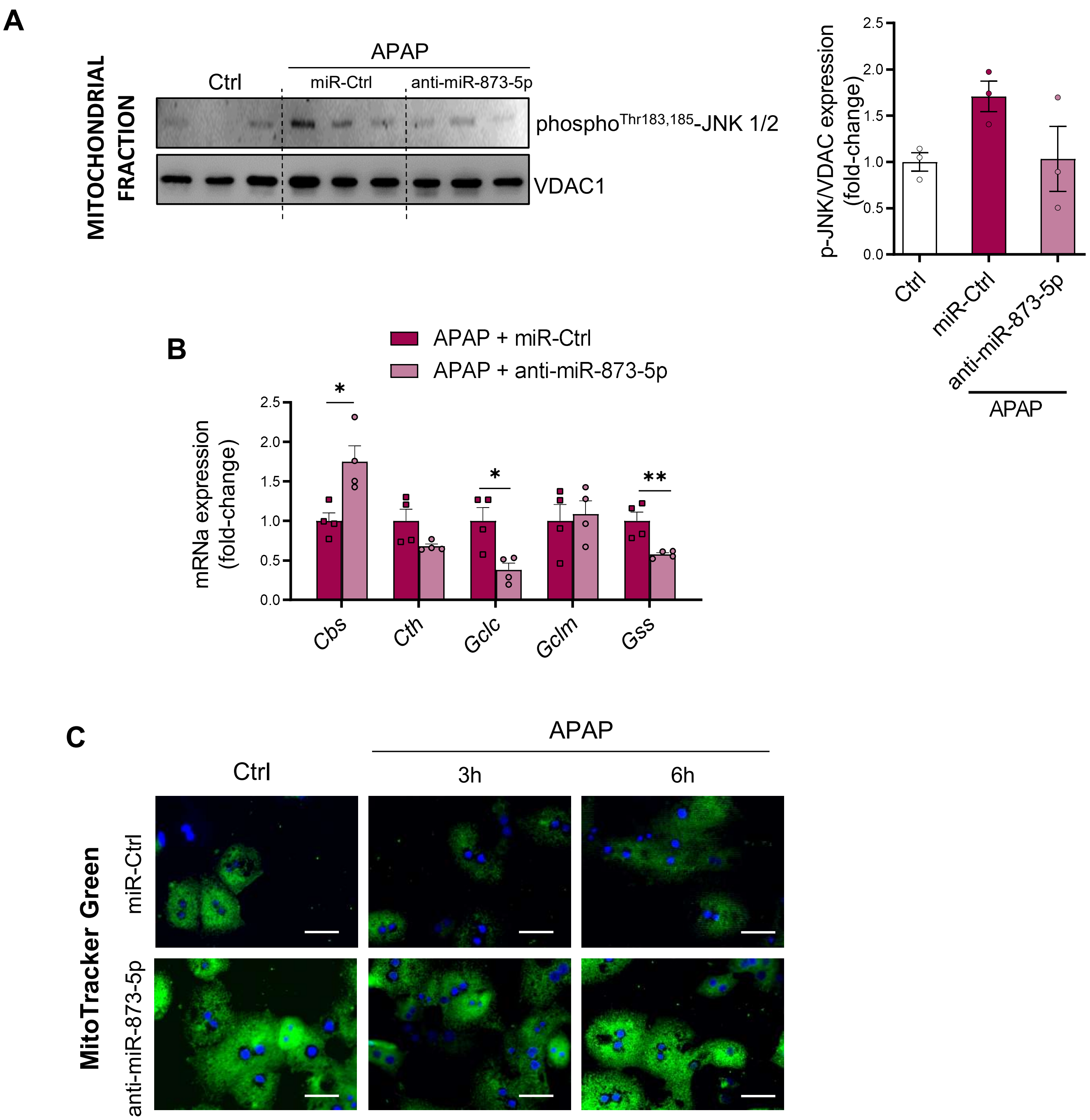

Figure S4. Reduced oxidative stress and mitocondrial dysfunction by anti-miR-873-5p **A.** Western blot determination of phosphorylated c-Jun N-terminal kinase (p-JNK) in mitochondria (VDAC was used as a loading control) and **B.** Relative mRNA expression of genes from transsulfuration pathway and glutathione synthesis in liver from mice treated with 360 mg/kg APAP for 48 h and 24 h of a treatment with anti-miR-873-5p or miR-Ctrl; **C.** Representative micrographs of MitoTracker Green staining in primary hepatocytes treated with acetaminophen (APAP) for 3 and 6 hours and either anti-miR-873-5p or miR-Ctrl. Scale bar corresponds to 50 µm. **p*<0.05, ***p*<0.01. (Related to Figure 4)

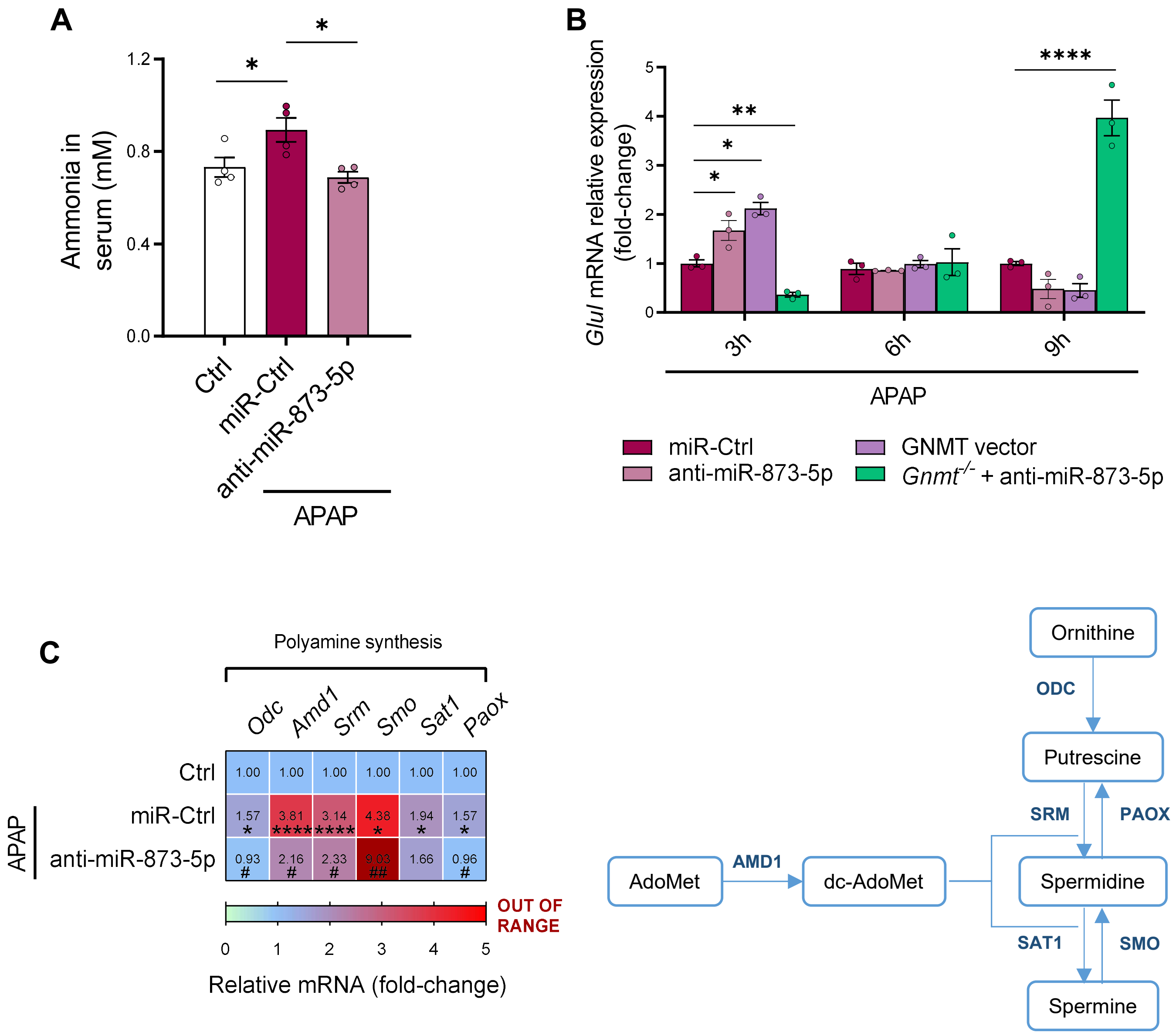

Figure S5. Ammonia homeostasis and polyamine synthesis upon anti-miR-873-5p knockdown **A.** Ammonia measurement in serum from mice treated for 48 h with 360 mg/kg acetaminophen (APAP) and an anti-miR-873-5p or miR-Ctrl; **B.** Relative glutamine synthetase (*Glul*) mRNA expression in wild type or glycine N-methyltransferase-lacking (*Gnmt*$^{-/-}$) treated with 10 mM APAP for 3, 6 and 9 h and transfected with an anti-miR-873-5p or miR-Ctrl. *p<0.05 and **p<0.01 are shown ; **C.** Relative mRNA expression of genes from polyamine synthesis, and schematic representation, in liver from mice administered 360 mg/kg APAP for 48 h and treated with either anti-miR-873-5p or miR-Ctrl. *p<0.05; ****p<0.0001 versus Ctrl; $^{\#}p$<0.05, $^{\#\#}p$<0.01, vs APAP + miRCtrl are shown. (Related to Figure 5)

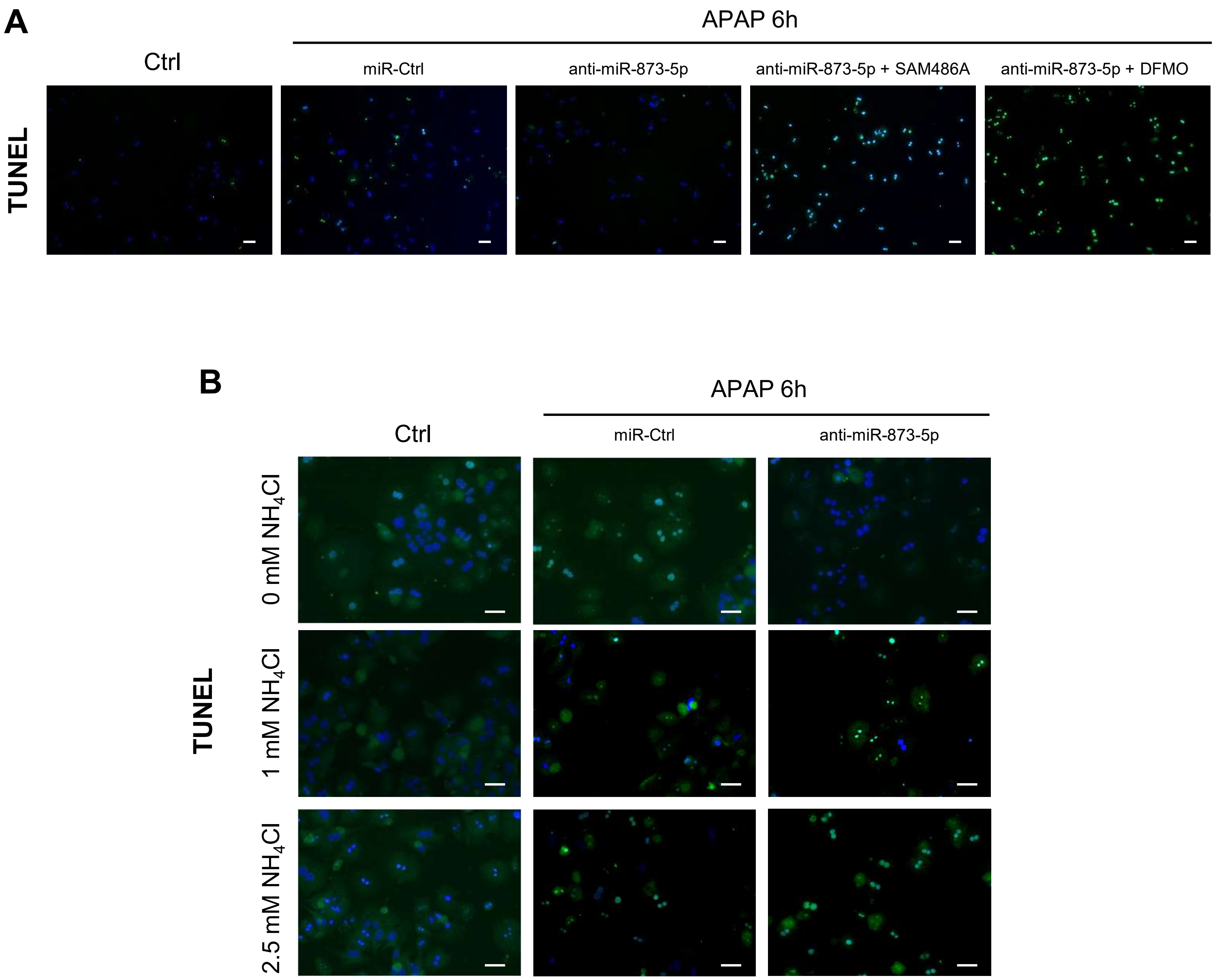

Figure S6. Representative micrographs of TUNEL staining under high ammonia concentrations and polyamine synthesis inhibitors. Representative micrographs of TUNEL staining of primary hepatocytes treated for 6 h with 10mM APAP, an anti-miR-873-5p or miR-Ctrl and either: **A.** different concentrations of ammonium chloride (1mM and 2.5mM NH4Cl); or **B.** 1 μM SAM486A or 0.5 μM DFMO. Scale bar corresponds to 50 μm

Table S1. List of antibodies used for immunohistochemistry.

REAGENT	PROVIDER	ID	SOURCE	DILUTION
Cyclin D1 (92G2)	CST	2978	Monoclonal IgG produced in Rabbit	1/100
F4/80 (Cl: A3-1)	Bio-Rad	NCA497bb	Monoclonal IgG2b produced in Rat	1/50
Glutamine synthetase (GLUL)	Novus Biological	NB110-41404	Polyclonal IgG produced in Rabbit	1/100
Proliferating cell nuclear antigen (PCNA) (F-2)	Santa Cruz Biotechnology	sc-25280	Monoclonal IgG2a produced in Mouse	1/100

Table S2. List of primers used for RT-qPCR.

Gene	Gene	Species	Forward 5´-3´	Reverse 5´-3´
ADP Ribosylation Factor Related Protein 1	*ARP/Arp*	Hs Mm	CGACCTGGAAAGTCCAACTAC	ATCTGCTGCATCTGCTTC
Glycine N-methyltransferase	*Gnmt*	Hs	AGTACAAGGCGTGGTTGCTT	ATCTTTGTCCAGCGTCAACC
Adenosylmethionine Decarboxylase 1	*Amd1*	Mm	TACACCCCAGGTGAGAGTTTG	CTTCCACAGAAGTTCATGCCAA
Arginase	*Arg*	Mm	TGTGAAGAACCCACGGTCTG	GCACCACACTGACTCTTCCA
Argininosuccinate Lyase	*Asl*	Mm	AAGTGGAGCCCTGAAGAAACC	AAATCCCCCAGCCCACTCAT
Argininosuccinate Synthase 1	*Ass1*	Mm	TCCAGTGCACTCTACGAGGA	CAATCTCCACCTGTCTGCGA
Cysthationine beta synthease	*Cbs*	Mm	CTTCAGGGACATCCCAGTGT	AGCTGCCAGGTACATCTGCT
C-C Motif Chemokine Ligand 5	*Ccl5*	Mm	GCTGCTTTGCCTACCTCTCC	TGCAGTGACAAACACGACTGC
Carbamoylphosphate synthase 1	*Cps1*	Mm	TCAGCCTACAGCCTCAACTG	TGTCCAATTTGTTTGTAACCAGTGT
Cysthationine gamma-lyase	*Cth*	Mm	GCAATGGAATTCTCGTGCCG	GCAGCCACTGCTTTTTCCAA
C-X-C Motif Chemokine Ligand 1	*Cxcl1*	Mm	GGTGTCCCCAAGTAACGGAG	TTGTCAGAAGCCAGCGTTCA
Cyclin B	*Cyclin B*	Mm	TCTTGACAACGGTGAATGGACAC	ATGCAGCACCTGGCTAAGAATG
Cyclin E	*Cyclin E*	Mm	TGTTTAACATGATCCTCCAAACC	GAAATTGCCAAGATTGACAAGAC
CyclinD1	*Cyclin D1*	Mm	TCAGTGTGACCCGGACTG	CCTTGGGGTCGACGTTCTG
Glyceraldehyde-3-Phosphate Dehydrogenase	*Gapdh*	Mm	TGAAGCAGGCATCTGAGGG	CGAAGGTGGAAGAGTGGGAG
Glutamate-Cysteine Ligase Catalytic Subunit	*Gclc*	Mm	TGCAGGAGCAGATTGACAGG	TAGAGAAAGCAAGCGGGTGG
Glutamate-Cysteine Ligase Modulatory Subunit	*Gclm*	Mm	GCTGAGGTACTCGGTCATCG	GCTGGAGTTAAGAGCCCCTC

Table S2. List of primers used for RT-qPCR.

Gene	Gene	Species	Forward 5´-3´	Reverse 5´-3´
Glutaminase 1	*Gls1*	Mm	TGCGAACATCTGATCCCAGG	AGCATGACACCATCTGACGTT
Glutaminase 2	*Gls2*	Mm	ATCTTAGCCAGGACACGCTG	AGGGGAGAAAGAGAACGACT
Glutamine synthetase	*Glul*	Mm	CTCTCCCAGCTCTTCCCTTT	TGCATACCCGATGAGATGAA
Glycine N-methyltransferase	*Gnmt*	Mm	ACCAGTATGCAGATGGGGAG	CCAATTGTCAAAGGATGGCT
Glutathione Synthetase	*Gss*	Mm	GACAACCCCTACCCTGTGTG	TGGAAGAGACAAGCTCCCCT
Homooxigenase-1	*Homo-1*	Mm	AAGCTTTTGGGGTCCCTAGC	ACAGCTGCTTTTACAGGCCA
Methionine Adenosyltransfe-rase 1A	*Mat1a*	Mm	GACACCATCAAGCACATTGG	ATGCATTCCTCGGTCTCATC
Methionine synthase	*Mtr*	Mm	TAGTATCGCCCAGGCTGACT	TCTCCTCAGCGGCTTTTCTG
Methylenetetra-hydrofolate Reductase	*Mthfr*	Mm	CGTGCACGATGTGGTAGTTG	CCATCCTCAGACCCTGTTGT
Methenyltetra-hydrofolate Synthetase	*Mthfs*	Mm	CCACTGGTGGACTTGACCTC	AAGCCATGGTGTAGGGCTTC
Ornithine decarboxylase	*Odc*	Mm	CTGTGCTTCTGCTAGGATCAATGT	GCCTTAATTCAAGCTAAACTTGCA
Ornithine transcarbamilase	*Otc*	Mm	GTCATTAGTGTTCCCAGAGGCA	TGAGTAGTCTGTCAGCAGGGA
Polyamine Oxidase	*Paox*	Mm	TAGAGTGTTGTGTGAGCGGC	CAGCCAAGATGCAATCCAGC
Proliferating Cell Nuclear Antigen	*Pcna*	Mm	TACAGCTTACTCTGCGCTCC	TTGGACATGCTGGTGAGGTT
Spm/Spd N1-Acetyltransferase	*Sat1*	Mm	GCGCAGTTTCCCCGAAGTA	GGGAGCGTCCTCTTCTCAGT
Spermine Oxidase	*Smo*	Mm	TTGTGCTCATCACCTTCAGC	TGCCAAACATGGCAAATAGA
Spermidine Synthase	*Srm*	Mm	GTGGTGGACTACGCCTACTG	TGGTGCTCGGGTTTTTGCTA
β-catenin	*β-catenin*	Mm	GCTGATTTGATGGAGTTGGAC	AGGAGCTGTGGTGGTGGCACCAGAATGGAT

Table S3. List of antibodies used for western blot

REAGENT OR RESOURCE	PROVIDER	IDENTIFIER	SOURCE	Dilution
β-Catenin	Cell Signaling Technology	9562S	Polyclonal IgG produced in Rabbit	1/2000
Cyclin D1	Cell Signaling Technology	2978S	Monoclonal IgG produced in Rabbit	1/1000
GNMT	Homemade	-	Polyclonal IgG produced in Rabbit	1/5000
Histone H3	Santa Cruz Biotechnology	sc-518011	Monoclonal IgG3 produced in mouse	1/1000
PCNA (F-2)	Santa Cruz Biotechnology	sc-25280	Monoclonal IgG2a produced in Mouse	1/2000
P-JNK1/JNK2 (Thr183, Tyr185)	ThermoFisher Scientific	44-682G	Polyclonal IgG produced in Rabbit	1/1000
VDAC1/Porin + VDAC3 [20B12AF2]	Abcam	ab14734	Monoclonal IgG2b produced in Muse	1/1000
Anti-mouse, HRP-linked secondary	Cell Signaling	7076S	IgG produced in Horse	1/5000
Anti-rabbit, HRP-linked secondary	Cell Signaling	7074S	IgG produced in Goat	1/5000

Table S4. List of primers used for micro-RNA specific RT-qPCR.

RNA	Species	Sequence 5´-3´	Reference
U6 snRNA	Hs<br>Mm	GTGCTCGCTTCGGCAGCACATATACTAAAATTGGAACGA<br>TACAGAGAAGATTAGCATGGCCCCTGCGCAAGGATGAC<br>ACGCAAATTCGTGAAGCGTTCCATATTTT	Cat. # 4427975<br>ID: 001973
miR-873-5p	Hs<br>Mm	GCAGGAACUUGUGAGUCUCCU	Cat. # 4427975<br>ID: 002356
